# Gestational stage affects amniotic epithelial cells phenotype, methylation status, immunomodulatory and stemness properties

**DOI:** 10.1007/s12015-014-9519-y

**Published:** 2014-05-28

**Authors:** Barbara Barboni, Valentina Russo, Valentina Curini, Alessandra Martelli, Paolo Berardinelli, Annunziata Mauro, Mauro Mattioli, Marco Marchisio, Patrizia Bonassi Signoroni, Ornella Parolini, Alessia Colosimo

**Affiliations:** 1 Faculty of Veterinary Medicine, University of Teramo, Piazza A. Moro 45, 64100 Teramo, Italy; 2 Department of Biomorphology, University of Chieti, Chieti, Italy; 3 Centro di Ricerca E. Menni, Fondazione Poliambulanza - Istituto Ospedaliero, Brescia, Italy; 4 Stem TeCh Group, Chieti, Italy

**Keywords:** Amniotic epithelial cell, Sheep, Gestational age, In vitro plasticity, Immunomodulatory activity, Global DNA methylation

## Abstract

Stem cells isolated from amniotic epithelium (AECs) have shown great potential in cell-based regenerative therapies. Because of their fetal origin, these cells exhibit elevated proliferation rates and plasticity, as well as, immune tolerance and anti-inflammatory properties. These inherent attitudes make AECs well-suited for both allogenic and xenogenic cellular transplants in animal models. Since in human only at term amnion is easily obtainable after childbirth, limited information are so far available concerning the phenotypic and functional difference between AECs isolated from early and late amnia. To this regard, the sheep animal model offers an undoubted advantage in allowing the easy collection of both types of AECs in large quantity. The aim of this study was to determine the effect of gestational age on ovine AECs (oAECs) phenotype, immunomodulatory properties, global DNA methylation status and pluripotent differentiation ability towards mesodermic and ectodermic lineages. The immunomodulatory property of oAECs in inhibiting lymphocyte proliferation was mainly unaffected by gestational age. Conversely, gestation considerably affected the expression of surface markers, as well the expression and localization of pluripotency markers. In detail, with progression of gestation the mRNA expression of *NANOG* and *SOX2* markers was reduced, while the ones of *TERT* and *OCT4A* was unaltered; but at the end of gestation NANOG, SOX2 and TERT proteins mainly localized outside the nuclear compartment. Regarding the differentiation ability, *LPL* (adipogenic-specific gene) mRNA content significantly increased in oAECs isolated from early amnia, while *OCN* (osteogenic-specific gene) and *NEFM* (neurogenic-specific gene) mRNA content significantly increased in oAECs isolated from late amnia, suggesting that gestational stage affected cell plasticity. Finally, the degree of global DNA methylation increased with gestational age. All these results indicate that gestational age is a key factor capable of influencing morphological and functional properties of oAECs, and thus probably affecting the outcome of cell transplantation therapies.

## Introduction

Amniotic epithelial cells (AECs) are formed from epiblast before gastrulation and have been shown to retain a remarkable and evolutionary conserved plasticity, as well as, to possess high self-renewal capacity [1–5]. Moreover, being the placenta the site in which foetus-maternal immune tolerance is played, these cells conserve immunomodulatory properties [6], thus being well tolerated after allo- and xeno-transplantation [7–10]. In addition, in long-term transplantation experiments, amniotic tissues and cells [11, 12], including amniotic epithelial cells (AECs) [13, 14] did not cause any evidence of tumorigenesis, thus demonstrating the safety of this stem cell source.

In humans, due to the wide availability of gene sequences and specific antibodies, AECs have been extensively studied to date, demonstrating that they express several stem cell-specific markers, such as Stage Specific Embryonic Antigen 3 (SSEA-3) and Stage Specific Embryonic Antigen 4 (SSEA-4) [15], and specific pluripotency markers, including Octamer-binding protein 4 (OCT-4), SRY-related HMG-box gene 2 (SOX2), Homeobox protein NANOG (NANOG), Tumor rejection antigen (TRA 1–60), Tumor rejection antigen (TRA 1–81), Left-right determination factor 2 (LEFTY2), Fibroblast growth factor type 4 (FGF4), and Reduced expression 1 (REX1) [15–17]. Among them, OCT-4, NANOG and SOX2 represent the main transcription factors that guarantee the maintenance of self-renewal and pluripotency properties [15, 17]. In addition, several evidences have demonstrated that AECs express epithelial and mesenchymal specific markers [18, 19], providing a clinical use of these cells for both tissue lineages [3, 16, 20, 21]. More recently, AECs have been shown to be able to differentiate into cells expressing germ cell specific markers [22] and to differentiate into granulosa cells for restoring folliculogenesis in a mouse model of premature ovarian failure [23].

The phenotype of human AECs includes also SSEA1, CD34 and CD133 molecular markers, while CD117 (c-kit) and CCR4 may be poorly present or not expressed at all [1, 15].

In sheep, several of the previously described antigens and pluripotency associated transcription factors have been confirmed to be expressed [3, 20], thus showing a conserved behavior of this class of amniotic-derived cells amongst medium-sized mammals. This important feature allowed the use of ovine AECs (oAECs) in experimentally-induced defects to evaluate their regenerative properties and safety on an animal model of high translational value, in particular, for muscle skeleton diseases [3, 24, 25].

A conserved phenotype and expression profile of stemness markers were, firstly, demonstrated by Mattioli et al. [3] regarding in particular the pluripotency associated transcripion factors OCT-4, SOX-2 and NANOG; on the contrary, TERT expression was detected in sheep AECs but not in the human counterparts [15]. The study of Mattioli et al. [3] had also demonstrated the in vitro and in vivo function exerted by oAECs in sustaining osteogenic tissue lineage regeneration, suggesting an alternative cell source for repairing skeletal tissues disease. Subsequently, Barboni at al. [9] confirmed the oAECs mesodermal stemness properties in an experimentally-induced tendon defect, extending the regenerative properties of this type of amniotic-derived cells to a tissue that cannot spontaneously repair. This result demonstrated also the safety of this type of amniotic-derived cells in a short time allo-transplantation experiment.

The oAECs phenotype and the encouraging results obtained on an in vivo experimental model indicate that these cells represent a good candidate as stem/progenitor cells to develop cell-based regenerative protocols. However, scarce information on their biological properties and plasticity, due to the effect of the gestational stage, are so far available.

Albeit limited, some evidences support the idea of a morphological and functional change during gestation of the amniotic tissue and the derived stem cells. A study conducted by Shandley et al. [26] described the morphology evolution of the ovine amnion during gestation. This study showed that, with the progress of gestation, cells grow and assume a more cuboidal morphology. At the same time, there is an increase of the number of cytoplasmic components, as lipid droplets and vesicles, while rough endoplasmic reticulum tends to organize itself in the form of bags, instead of tubules. Moreover, in the early stages, the apical microvilli appeared irregular in shape, size and frequency, while at the end of pregnancy they appeared shorter and more frequent. Also, an increase of appositions tips was observed. The microstructure of amniotic membrane in the early stages of gestation seems to show an increased permeability compared to stages that are older than 100 days of gestation. This behavior of ultrastructure of amnion and AECs observed in sheep in relation to the time of gestation was also identified in human, monkey, and cattle [27–29].

In addition, some relevant molecular expression profiles seem to be modified during pregnancy. In fact, Nakajima et al. [30] demonstrated a reduction of telomerase activity in rat AECs collected at the end of gestation, while Izumi et al. [31] showed that there is a reduced expression in stem cell markers in human term amnion compared to fetal ones.

In the present study, the in vitro behavior of oAECs derived from different stages of gestation was analytically described. The sheep model offers, in fact, the possibility of having a large number of oAECs at defined gestational stages.

Cells isolated from amnia derived from fetuses at early and late stages of pregnancy were compared in their phenotype, immunomodulatory properties, in vitro plasticity, and status of global DNA methylation. In particular, oAECs isolated from amnia of sheep fetuses of 4–6 cm (early-gestation: <1.5 month) and of 40–45 cm (late gestation: 5 months) of length [32] were evaluated. Both categories of cells were analyzed before and after their in vitro differentiation towards two mesodermal tissue lineages (adipogenic and osteogenic) and an ectodermal one (neurogenic), that were previously documented either in human and ovine amniotic derived cells, respectively [15, 20].

## Materials and methods

### Ethics statement

All cells and tissues were collected from animals slaughtered at the local abattoirs for food purposes (Chieti, Pianella); for this reason this study did not require any ethics statement. The collection from the slaughterhouse of biological material is normed by specific conventions subscribed between the Faculty of Veterinary Medicine of Teramo and the ASL, organ of the Ministry of Health vacated to the control and safety of slaughterhouses. In each slaughterhouse the collection of tissues is controlled by an inspector which is a Veterinary that follows the regularity of all the procedures.

### Isolation and culture of oAECs

Sheep amnia were collected at a local abattoir from 2 to 3 years old sheep of Appeninica breed and with a mean weight of 45 + 6 Kg by removing the whole pregnant uterus and bringing it at 30 °C to the laboratory in maximum 1 h, for further evaluation and processing. Four amnia derived from fetuses of 4–6 cm (from 1 to 1.5 months of gestation) and four amnia from fetuses of 40–45 cm of length (term gestation), according to Barone [32], were used. Once the uterus wall was opened, oAECs were collected from the amniotic membrane. This latter was mechanically peeled off in order to obtain pieces of amniotic membrane of approximately 3–5 cm. Membrane pieces were washed in Phosphate Buffered Saline (PBS; Sigma) and incubated in 0.25 % Trypsin/EDTA 200 mg/L at 37.5 °C for 20 min. The cell debris released during this first digestion was discarded. The trypsin incubation then proceeded for further 30 min under gentle agitation. Subsequently, cell suspension was collected, filtered through a 40 μm cell filter and poured into a 50 ml falcon tube containing Fetal Calf Serum (FCS) (Lonza) at a final concentration of 10 % to inactivate trypsin. Each falcon tube was centrifuged and the pelleted vital cells were counted after trypan-bleu staining by using a haemocytometric chamber. Generally 2 × 10^5^ cells were seeded in 250 ml flasks in growth medium (GM) composed of Minimum Essential Medium α − transformation (α − MEM) (Gibco) supplemented with 20 % FCS, 1 % Ultraglutamine (Lonza), 1 % Penicillin/Streptomycin (Lonza) and 10 ng/ml Epidermal Growth Factor (EGF) (Sigma) [3]. The cells were incubated at 38.5 °C in 5 % CO_2_. After 7 days of culture, the medium was replaced with fresh one, and was subsequently replaced 3 times a week. At 80 % confluence, the flasks were washed to remove dead cells and debris, the cells were harvested with 0.05 % trypsin-EDTA for 5 min at 38.5 °C and plated again at 3 × 10^3^/cm^2^. At 70 % of confluence, cells were then used for stemness characterization and for differentiation experiments. These experiments were performed on freshly adhered oAECs cultured as described above.

The proliferative activity of oAECs was analyzed at different passages by calculating the population doubling time (i.e. the time during which the cell population doubled), as previously described [33].

Differently, in order to compare the immunomodulatory properties and stability of oAECs collected at different stages of gestation, their inhibitory role on lymphocyte proliferation was tested by using either freshly adhered cells or oAECs after three passages of expansion.

To this aims, when adhered cells reached 70–80 % of confluence dead cells and debris were removed with the medium, while the oAECs were dissociated with 0.05 % trypsin-EDTA and plated again at 3 × 10^3^/cm^2^ for three consecutive expansion passages.

### Immunomodulatory tests

The effect of oAECs from early or late pregnancy, both at culture passage 1 (P1) and passage 3 (P3), on peripheral blood mononuclear cells (PBMNCs) proliferation was studied as previously reported for amniotic cells derived from other species [34, 35].

Briefly, ovine PBMNCs were obtained by density gradient centrifugation (Lymphoprep, Axis-Shield, Oslo Norway) of 15 ml peripheral blood, as previously described [36].

Lymphocyte proliferation was obtained by activating PBMNCs (2 × 10^5^ PBMNC/well) through addition of phytohemagglutinin (PHA; Sigma) at a final concentration of 2 μg/ml.

To study the effects of oAECs on lymphocyte proliferation in a cell-to-cell contact setting, different amounts of cultured oAECs (2 × 10^5^, 1 × 10^5^, 0.5 × 10^5^, 0.25 × 10^5^, 0.125 × 10^5^; 0.0625 × 10^5^) were plated in RPMI complete medium and left to adhere overnight. The next day, cells were γ-irradiated (3,000 cGy), and 2 × 10^5^ activated PBMNCs were added to each well, obtaining PBMNC:oAEC ratios of 1:1, 1:0.5, 1:0.25, 1:0.125 and 0.0625. oAECs were irradiated to ensure that any proliferation observed could be attributed solely to the proliferation of responder lymphocytes. PBMNCs in absence of oAECs were used as controls. All cultures were carried out in triplicate, using flat-bottomed 96-well tissue culture plates (Corning-Celbio), in a final volume of 200 μl of RPMI complete medium.

For lymphocyte proliferation tests with oAECs added not in cell-to-cell contact, transwell chambers with 0.4-μm pore size membranes (Corning-Celbio) were used to physically separate the lymphocytes from the oAECs plated in the upper chambers of the transwell inserts at the same concentration reported above. The next day, 2 × 10^5^ PBMNCs plus PHA were added to each lower chamber, obtaining PBMNC: oAEC ratios of 1:1, 1:0.5, 1:0.25, 1:0.125, and 0.0625.

In all cases, lymphocyte proliferation was assessed after 3 days of culture by adding 0.67 μCi per well of [^3^H]-thymidine (INC Biomedicals) for 16–18 h. Cells were then harvested with a Filtermate Harvester (PerkinElmer), and thymidine incorporation was measured using a microplate scintillation and luminescence counter (Top Count NXT; PerkinElmer).

### Differentiation potential

To evaluate oAECs plasticity, cells isolated from both categories of early and term amniotic membranes were differentiated into adipogenic, osteogenic and neurogenic cell lineages.

For the induction of adipogenic differentiation, cells at 70 % of confluence were cultured for 30 days in (DM) represented by supplementing GM with 10 % FCS, 2 mM Ultraglutamine, 0.1 μM Dexamethasone, 0.45 mM 3-isobutyl-1-methyxanthine, 0.2 mM indomethacin, 1 μM rosiglitazone, 10 μg/ml insulin [37]. The differentiation was assessed by Oil Red O and RT-PCR for a previously identified [21] adipogenic-specific marker, the lipoprotein lipase (*LPL*).

Oil Red O assay was performed by staining cells for 15 min with fresh Oil-Red O solution (Sigma) (three parts of a stock solution 0.5 % in isopropanol and two parts of distilled water) and washing them three times with distilled water. The reaction was evaluated under an optical microscope. Osteogenic differentiation was performed at 70 % of confluence. Cells were cultured for 21 days in DM containing 10 % FCS, 50 μM ascorbic acid, 10 mM β-glycerophosphate, and 0.2 μM dexamethasone. Extracellular matrix mineralization was assessed by Alizarin Red Staining, while osteogenic tissue lineage transformation was documented by analyzing an osteoblast-specific marker, osteocalcin (*OCN*) [3, 21]. Tissue specific genes are summarized in Table 1.

**Table 1:** Primer sequences used for RT-PCR

Gene	Accession No.	Primer sequences	Product size (bp)	PCR cycles
*NEFM*	FJ427307.1	F: 5′-ACCGACAGCCCTCCATCGCC-3′	156	40
	Ovine	R: 5′-TGATGGCCGTCAGGGCTTCCT-3′		
*LPL*	NM_001009394.1	F: 5′-GTCACGGGCCCAGCAGCATT-3′	313	40
	Ovine	R:5′-GCCAGGTGACCCCCTGGTGA-3′		
*OCN*	DQ418490.1	F: 5′-AGACACCATGAGAACCCCCAT-3′	234	40
	Ovine	R: 5′-TTGAGCTCACACACCTCCCT-3′		
*NANOG*	FJ970651.1	F: 5′-TGGATCTGCTTATTCAGGACAG-3′	209	45
	Ovine	R: 5′-TGCTGGAGACTGAGGTATTTC-3′		
*TERT*	EU139125.1	F: 5′-TTGTCCCCGCAGGTGTCTTG-3′	176	45
	Ovine	R: 5′-TGACCGTGTTGGGCAGGTAG-3′		
*SOX2*	X96997.1	F: 5′-ACCAGAAGAACAGCCCGGAC-3′	264	45
	Ovine	R: 5′-TCATGAGCGTCTTGGTTTTCCG-3′		
*OCT4A*	NM_174580.1	F: 5′-TATGACTTGTGTGGAGGGATG-3′	327	45
	Bovine	R: 5′-AAAGAGAACCCCCAGGGTGA-3′		
*GAPDH*	AF030943.1	F: 5′-CCTGCACCACCAACTGCTTG-3′	224	40
	Ovine	R: 5′-TTGAGCTCAGGGATGACCTTG-3′		

For the neural differentiation, cells at 70 % of confluence were cultured for 30 days in differentiation medium (DM) composed of α − MEM with 10 % FCS, 2 mM Ultraglutamine, 1 % MEM non-essential amino acid solution 100X (Sigma), 55 μM 2-mercaptoethanol, 1 mM sodium pyruvate, 1 % Penicillin/Streptomycin, 5 × 10^−5^ M all-*trans* retinoic acid, and 10 ng/ml fibroblast growth factor (FGF)-4 (Promega) according to Miki et al. [15]. The differentiation was assessed by immunocytochemistry and by RT-PCR evaluating the protein and mRNA expression of neurofilament-specific molecular markers (Neurofilament heavy subunit, NF200 and neurofilament medium neuropeptide, NEFM), that were previously validated in sheep (unpublished observations).

The use of two different markers was due to the unavailability of a commercial sheep antibody against NEFM protein and to the absence in Gene Bank of a sheep specific sequence for the *NF200* gene.

### Flow cytometry

oAECs were screened by flow cytometer for the CD31, CD45 CD29, CD49f, and CD166 surface molecules, as well as, for MHC I and MHC II antigens and for the pluripotency-associated markers TERT, SOX2, NANOG, as previously described in Mattioli et al. [3]. The primary antibodies used for the analysis were purchased as indicated in Table 2. Staining for flow cytometer was performed on 5 × 10^5^ cells/sample by incubating them with 100 μl of 20 mM ethylenediaminetetraacetic acid (EDTA) at 37 °C for 10 min. Cells were then washed in 3 ml of washing buffer (PBS, 0.1 % sodium azide and 0.5 % Bovine Serum Albumine, BSA) and centrifuged (4 °C, 400×*g*, 8 min). For surface antigens staining, cell samples were suspended in 100 μl washing buffer containing the appropriate amount of surface antibody; samples were incubated for 30 min at 4 °C in the dark. Tubes were washed (3 ml of washing buffer), centrifuged (4 °C, 400×*g*, 8 min) and cells were resuspended with 1 ml 0.5 % paraformaldehyde, incubated for 5 min at room temperature (RT), washed, centrifuged (4 °C, 400×*g*, 8 min) and stored at 4 °C in the dark until the acquisition.

**Table 2:** Details of primary antibodies used in flow cytometry analysis

Antigen (dilution)	Fluorescent probe	Company details	
Hemopoietic markers			
CD31 (1:10)	FITC	AbD Serotec	Oxford, UK
CD45 (1:10)	FITC	AbD Serotec	Oxford, UK
Adhesion molecole			
CD29 (1:100)		VMRD	Pullman, WA, USA
CD49f (1:100)		Beckman Coulter	Fullerton, CA, USA
CD166 (1:100)	FITC	Ancell	Cambridge, UK
MHC antigens			
Class I (1:50)		Novus Biologicals	Cambridge, UK
Class II HLA-DR (1:50)		Abcam	Cambridge, UK
Stemness markers			
SOX2 (1:100)		Abcam	Cambridge, UK
TERT (1:100)		Calbiochem	Gibbstown, NJ
NANOG (1:100)		Chemicon Int.	Billerica, MA

The un-conjugated primary antibodies were FITC marked by using Zenon Antibody Labelling Kit (Gibco, Invitrogen, Carlsbad, CA, USA), following the manufacturer’s instructions (Barboni et al., PlosOne 2012)

For intracellular antigens staining, cells were suspended in 1 ml of FACS Lysing solution (BD), vortexed and incubated at RT in the dark for 10 min. Samples were, then, centrifuged (4 °C, 400×*g*, 8 min); 1 ml of Perm 2 (BD) was added to each tube and cells were incubated at RT in the dark for 10 min. Samples were washed (3 ml of washing buffer) and centrifuged (4 °C, 400×*g*, 8 min). Cells were resuspended in 100 μl of washing buffer containing the appropriate amount of intracellular antibody and incubated for 30 min at 4 °C in the dark. Tubes were centrifuged (4 °C, 400×*g*, 8 min) and cells resuspended with 1 ml 0.5 % paraformaldehyde, incubated for 5 min at RT, washed, centrifuged (4 °C, 400×*g*, 8 min) and stored at 4 °C in the dark until the acquisition.

Finally, cells were analysed according to Barboni et al. [20] on a FACSCalibur flow cytometer (BD), using CellQuest™ software 3.2.1.f1 (BD).

Flow Cytometer Measurement was carried out by using as quality control Rainbow Calibration Particles (six peaks) and CaliBRITE beads (both from BD Biosciences).

Debris were excluded from the analysis by gating on morphological parameters (lymphocyte gate); 20.000 non-debris events in the morphological gate were recorded for each sample. All antibodies were titrated under assay conditions and optimal photomultiplier (PMT) gains were established for each channel. Data were analysed using FlowJo™ software (TreeStar, Ashland, OR). Mean Fluorescence Intensity Ratio (MFI Ratio) was calculated dividing the MFI of positive events by the MFI of negative events.

### Immunocytochemistry

The cells before and after differentiation were fixed by adding for 10 min PBS supplemented with 4 % paraformaldehyde. After washing with PBS, cells were permeabilized with 0.1 % Triton X-100 (PBS) for 10 min at RT. Nonspecific staining was blocked by incubating oAECs at RT in PBS/1 % BSA for 1 h. Cells were then incubated with the primary antibody (Ab) overnight at RT and then exposed to the secondary Ab for 40 min at RT.

For the global DNA methylation analysis, after cell fixation, samples were first denatured in 2 M HCl for 30 min at RT and then neutralized in 0.1 mol/L Tris HCl buffer (pH 8.5) for 10 min before primary antibody incubation. Nonspecific binding was blocked incubating the cells in normal goat serum (1:20 dilution; Sigma) for 1 h at RT. The primary antibody, a mouse anti-5-methylcytosine (1:500 in PBS containing 1 % BSA), was incubated overnight at 4 °C, and then with the secondary antibody for 1 h at RT.

All experiments with the omission of primary Abs were used as negative controls. Cell nuclei were identified with DAPI counterstaining.

All details on the primary and secondary Abs are shown in Table 3.

**Table 3:** Details of primary and secondary antibodies used for immunocytochemistry

Primary Abs (company details)	Primary Ab dilutions	Secondary Abs (company details)	Secondary Ab dilutions
Cytokeratin 8 (Abcam, Cambridge, UK)	1:200	Anti-Mouse Cy3 (Sigma-Aldrich, Missouri, USA)	1:500
NF200 (Chemicon Int. Billrerica, MA)	1:100	Anti-Mouse FITC (Sigma-Aldrich, Missouri, USA)	1:500
SOX2 (Calbiochem, Gibbstown, NJ)	1:200	Anti-Rabbit Alexa Fluor 488 (Invitrogen Ltd, Paisley, UK)	1:500
NANOG (Abcam, Cambridge, UK)	1:500	Anti-Mouse FITC (Sigma-Aldrich, Missouri, USA)	1:100
TERT (Chemicon Int. Billrerica, MA)	1:200	Anti-Mouse Cy3 (Sigma-Aldrich, Missouri, USA)	1:500
5-metyl-cytosine (Chemicon Int. Billrerica, MA)	1:200	Anti-Mouse Alexa Fluor 488 (Invitrogen Ltd, Paisley, UK)	1:750

Primary and secondary antibodies (Abs) are diluted in PBS supplemented with 1 % BSA

To study the cellular localization of pluripotency markers, at least 100 cells for each category of cell were analyzed. Results were recorded for statistical evaluation.

The degree of global DNA methylation was evaluated according to Russo et al. [38]. All samples were analyzed using an Axioskop 2 Plus incident-light fluorescence microscope (Carl Zeiss) equipped with a CCD camera (Axiovision Cam; Carl Zeiss) with a resolution of 1,300 × 1,030 pixels, configured for fluorescence microscopy, and interfaced to a computer workstation provided with an interactive and automatic image analyzer (Axiovision; Carl Zeiss). Digital images were acquired at ×400 magnification using standard filter set up for CY3 or DAPI. For quantitative purposes, digital images were consecutively captured immediately after immunoreactions. At the beginning of an imaging section, optimum exposure times were determined and held constant thereafter. Image acquisition was performed using a specific software (Axiovision; Zeiss). The images were imported in the 24-bit uncompressed TIF format, showing the staining pattern and the total fluorescence intensity (TFI) emitted by each nucleus. On the basis of the TFI emitted by each nucleus, it was possible to classify four degrees of fluorescent intensities (i.e., from 0 = no TFI to 3 = maximum TFI) [3, 39]. To study the degree of global DNA methylation of oAECs, at least 100 cells for each category of cell were analyzed. Results were recorded for statistical evaluation.

### Total RNA isolation and RT-PCR

RT-PCR analyses were performed on freshly isolated and differentiated oAECs in order to compare the expression profile of specific genes that are summarized in Table 1.

In detail, total mRNA was extracted by using TRI Reagent (Sigma) according to the manufacturer’s instructions. Integrity and size distribution were evaluated by 1 % agarose gel electrophoresis and ethidium bromide staining. Digestion of genomic DNA was carried out by DNaseI digestion (Sigma) for 15 min at room temperature. One microgram of total RNA of each sample was used for reverse transcription reaction with Oligo dT primer and BioScriptTM Kit (Bioline). 2X Ready mix™ Taq PCR Reaction mix (Sigma) was used for PCR reaction using 3 μl of cDNA and 0.5 μM of each primer, in a final volume of 25 μl. The primer sequences, Genbank number of reference mRNA sequence, product length and cycles are shown in Table 2. The reaction mixtures were incubated for 5 min at 95 °C, followed by 95 °C for 30 s, 55 °C for 30 s, 72 °C for 45 s and 72 °C for 7 min. For each gene, a reaction mixture with water instead of cDNA template was run at the same time as a PCR negative control. RT-PCR was normalized by the transcriptional levels of *GAPDH*. The PCR products were separated on 2 % agarose gel stained with ethidium bromide, visualized on a Gel Doc 2000 (Biorad) and analyzed with Quantity One 1-D Analysis software (Biorad). Each PCR reaction was carried out in triplicate.

### Statistical analysis

Data reported in this paper are the mean (±SD) of at least three independent experiments, each performed in triplicate. The data were checked for normal distribution by Shapiro-Wilks W test, and were compared by ANOVA test followed, if necessary, by post-hoc Tukey test (StatistiKL Version β). Student’s *t*-test was applied to assess differences between groups for the data related to immunomodulatory tests. The differences were considered significant for *p* values of <0.05.

## Results

### Morphology, immunomodulatory properties, phenotype, and pluripotency markers expression of oAECs collected from fetuses of different gestational stage

#### Morphology

After the enzymatic digestion, all freshly isolated oAECs displayed a typical poliedric shape and were confirmed to be epithelial by the immunocytochemistry analysis of cytokeratin 8, an epithelial specific lineage marker, as previously described [20] (data not shown).

Ovine AECs obtained from amniotic membranes of early stage fetuses (4–6 cm) adhered to the flask bottom in 4–6 days and displayed a higher self-renewal feature (doubling time of ~15 h) compared to oAECs derived from amnia of fetuses of 40–45 cm of length, that adhered in 7–10 days and proliferated with a mean doubling time of ~24 h.

#### Immunomodulatory properties

**Fig. 1:**
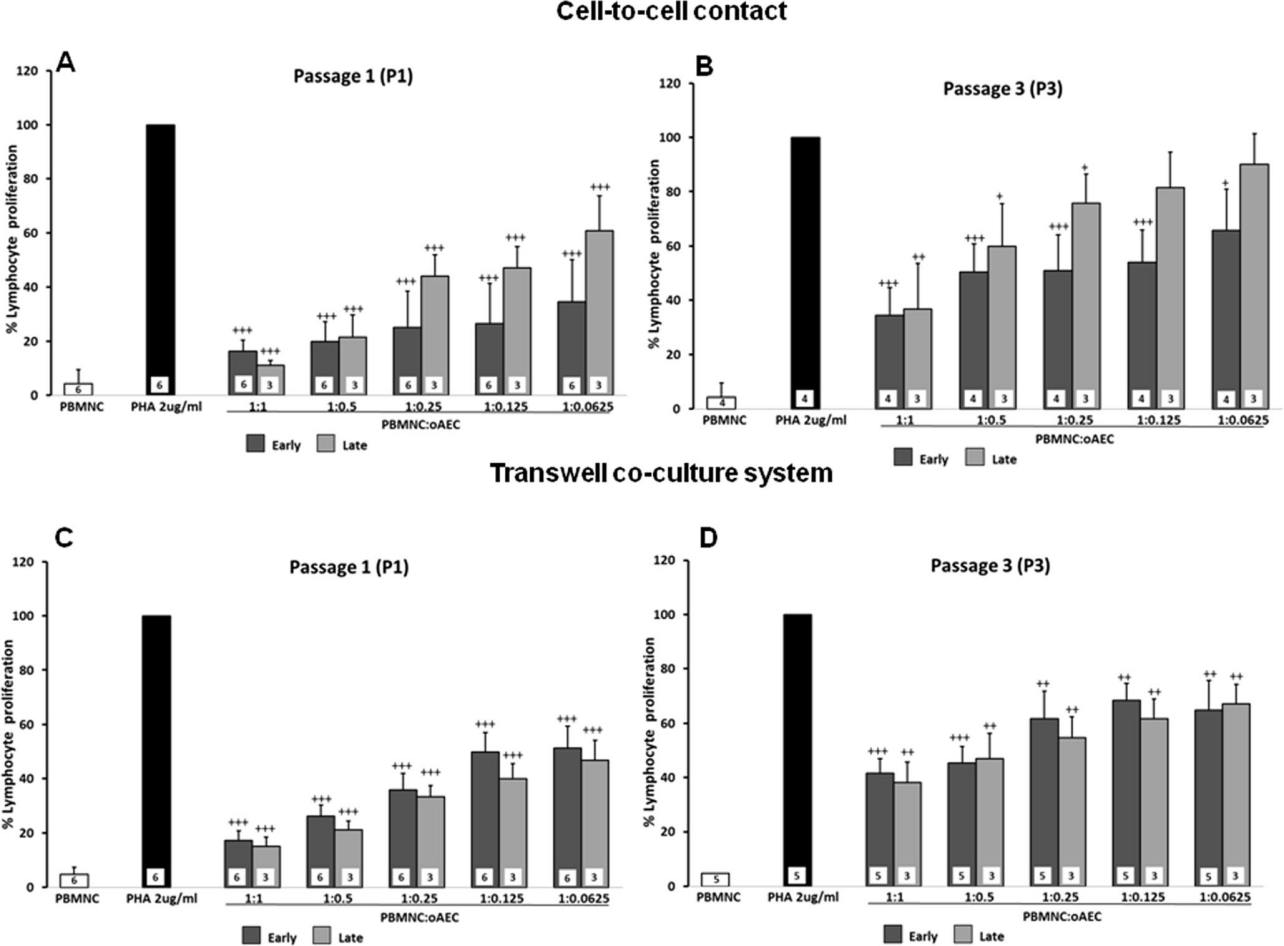
Effect of oAECs, harvested from amnia at different pregnancy times (early vs late), on the PHA-stimulated lymphocyte proliferation. oAECs harvested from early (*dark grey bars*) or late (*light grey bars*) pregnancy amnia were used at passage 1 (panels **a** and **c**) or passage 3 (panels **b** and **d**) of culture and put in contact with PBMNCs (panels **a** and **b**) or in a transwell system (panels **c** and **d**). oAECs were added to obtain different PBMNC:oAECs ratios (1:1, 1:0.5, 1:0.25, 1:0.125, and 0.0625). Data are represented as mean and SD. The number of the independent experiments performed is indicated inside the bars. ^**+**^ = *p* < 0.05, ^**++**^ = *p* < 0.01; ^**+++**^ = *p* < 0.001 versus PHA-stimulated PBMNCs (in absence of oAECs)

**Fig. 2:**
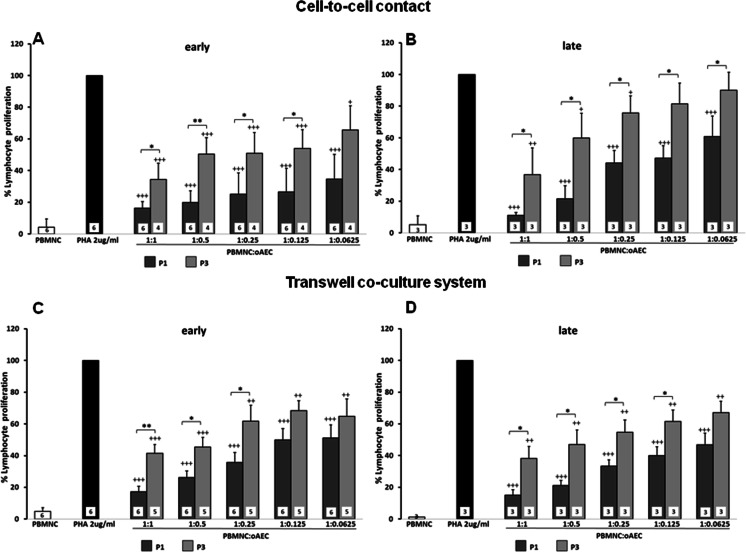
Effect of oAECs, at different culture passages (P1 vs P3), on the PHA-stimulated lymphocyte proliferation. “Early” and “late” oAECs were used at passage 1 (P1; *dark grey bars*) or at passage 3 (P3; *light grey bars*) of culture and were seeded in contact with PBMNCs (panels **a** and **b**) or in a transwell system (panels **c** and **d**). oAECs were added to obtain different PBMNC:oAEC ratios (1:1, 1:0.5, 1:0.25, 1:0.125, and 0.0625). Data are represented as mean and SD. The number of the independent experiments performed is indicated inside the bars. * = *p* < 0.05, ** = *p* < 0.01; oAECs at Passage 1 (*P1*) versus oAECs at Passage 3 (*P3*). ^+^ = *p* < 0.05, ^++^ = *p* < 0.01; ^+++^ = *p* < 0.001 versus PHA-stimulated PBMNCs (in absence of oAECs)

Ovine AECs obtained from early or late amnia were able to down-regulate PHA-induced lymphocyte proliferation both in cell-to-cell contact and physically separated from PBMNCs in a transwell system (Figs. 1 and 2).

Interestingly, in all cases, the inhibitory effect was dose dependent, and the higher effects were observed at a ratio of PBMNC:oAECs of 1:1, both in the cell-to-cell contact and transwell cultures (Figs. 1 and 2).

Concerning the immunomodulatory effect of oAECs isolated from amnia collected at early vs late pregnancy, it was not observed at passage 1 (P1) any significant difference in their ability in inhibiting lymphocyte proliferation, under both the test conditions (cell-to-cell contact and transwell system: Figs. 1a and b, respectively). Differently, passage 3 (P3) late oAECs were less effective: in contact they lost the in vitro immunomodulatory effect at 0.125 and 0.0625 dilution, while P3 early oAECs displayed still a significant inhibitory effect.

In addition, both early and late oAECs immunomodulatory abilities were negatively affected by culture passages (Fig. 1).

#### Phenotype

**Fig. 3:**
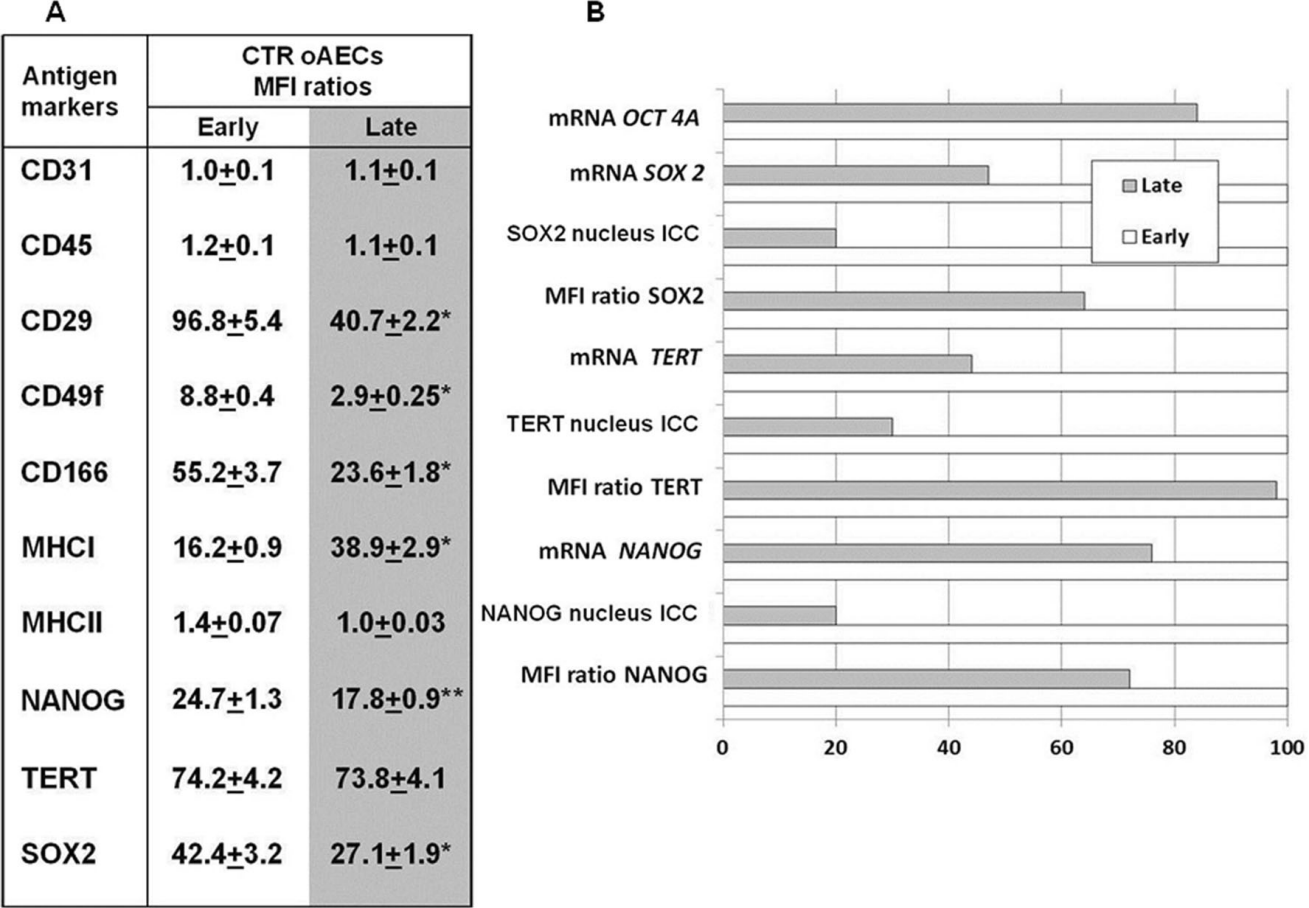
**a** Flow cytometry analysis of haemopoietic (CD31, CD45), adhesion molecules (CD29, CD49f and CD166), MHCI, MHCII and stemness (TERT, NANOG and SOX2) markers, expressed as Mean Fluorescence Intensity (*MFI*) ratios, in freshly isolated oAECs derived from early or late gestation amnia. * indicates significantly different values between early and late gestational amnia-derived oAECs (*p* < 0.05). ** indicate very significantly different values between early and late gestational amnia-derived oAECs (*p* < 0.01). **b** In the graph are shown mRNA levels (RT-PCR), protein expression (flow cytometry MFI Ratio) and nuclear localization (immunocytochemistry: *ICC*) of pluripotency markers. To study the differences of pluripotency markers between the two categories of cells, the data obtained from the early gestation oAECs were normalized to 100 % and compared to the relative value obtained by late gestation oAECs. The graph reveals that both the mRNA levels and the nuclear localization of all pluripotency markers were lower in oAECs isolated from late stages amnia

The stage of gestation affected cells phenotype; in fact, MFI ratios for the adhesion molecules (CD29, CD49f and CD166) were higher in oAECs collected from early amnia (*p* < 0.01; Fig. 3a), while the MHCI MFI ratio increased progressively with gestation (*p* < 0.01; Fig. 3a). Moreover, both categories of oAECs did not express haemopoietic markers (CD31, CD45), as well as, the MHCII antigen (Fig. 3a).

#### Pluripotency marker expression

Analogously, gestational age of oAECs affected the expression of the analyzed pluripotency markers both at protein and mRNA levels. In fact, cells isolated at the end of gestation displayed NANOG and SOX2 MFI ratios that were significantly lower (*p* < 0.01) than those recorded in oAECs isolated from fetuses at earlier stage of gestation (Fig. 3a). Only TERT MFI ratio was not affected by cell gestational age.

In addition, the majority of oAECs collected at early pregnancies (Fig. 3b) displayed TERT, NANOG and SOX2 within the nucleus as demonstrated by immunocytochemical analyses; on the contrary, only ~10–20 % of oAECs isolated from late pregnancies maintained the nuclear localization of pluripotency associated markers (Fig. 3b).

**Fig. 4:**
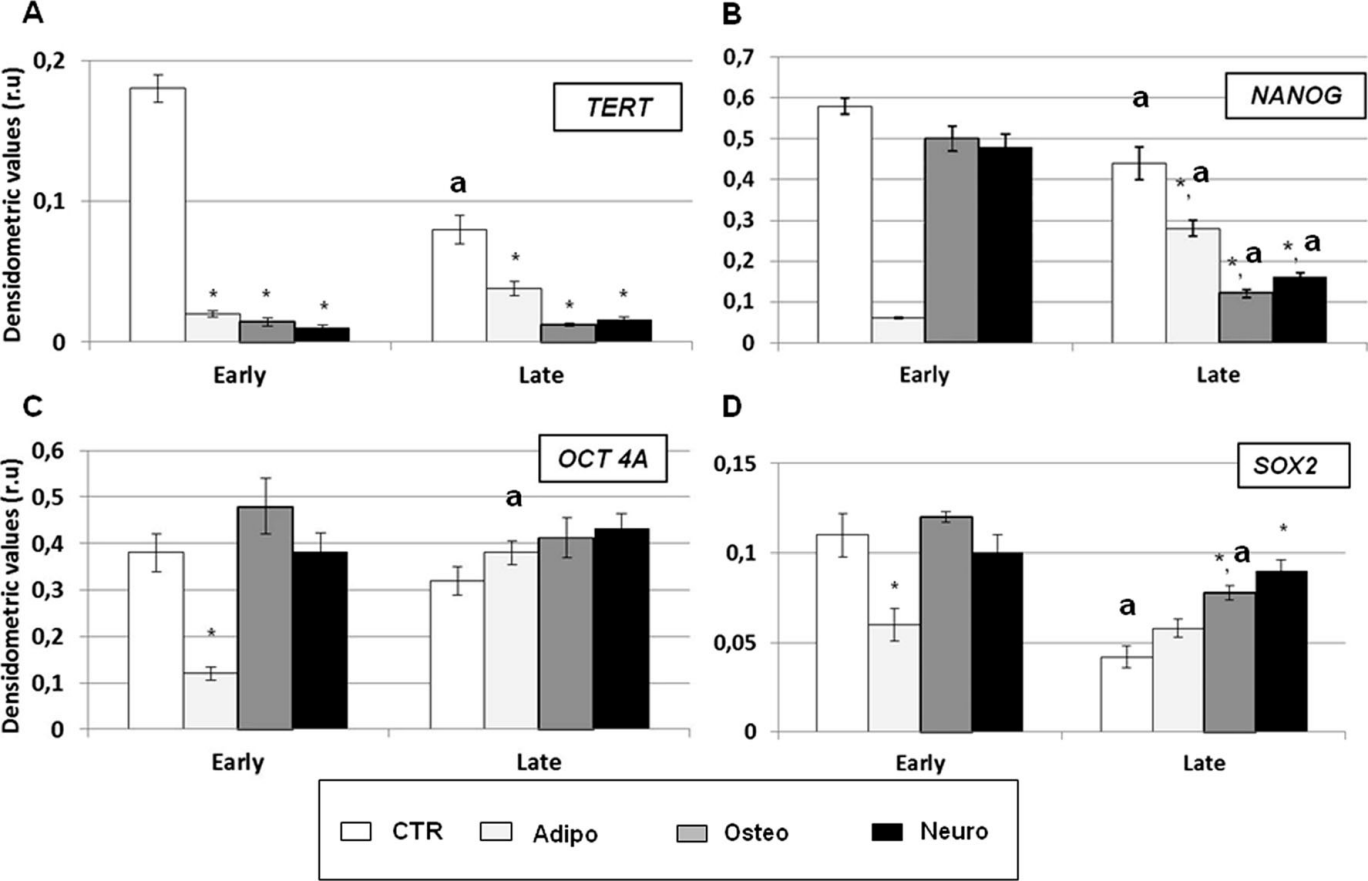
Expression profile of pluripotency markers in oAECs isolated from early or late gestational amnia. The mRNA content of *TERT, NANOG*, *OCT4A* and *SOX2* was analyzed in freshly isolated oAECs or in differentiated oAECs towards the adipogenic, osteogenic or neurogenic lineages (**a–d**) by RT-PCR. The semi-quantitative analyses of mRNA levels were normalized for *GAPDH* gene and expressed as mean of 3 different replicates ± SD. * significantly different values between freshly and differentiated oAECs within each cell category (*p* < 0.05); ^a^ significantly different values between oAECs isolated from early and late gestational age amnia, calculated within a similar category

The mRNA content of the pluripotency antigens were concordant with the relative protein profile (Figs. 3b and 4a–d). In fact, *NANOG*, *TERT* and *SOX2* mRNA levels resulted lower in oAECs isolated from late amnia, similarly to the corresponding proteins (Figs. 3b and 4a, b, d). Only *OCT4A,* that was detected exclusively by RT-PCR [40] for the absence of a commercial sheep antibody able to discriminate among the three main isoforms (OCT4A, OCT4B, OCT4B1), resulted quite similar in both cell categories (Figs. 3b and 4c).

In detail, in differentiated early or late oAECs, the *TERT* mRNA content underwent to a generalized decrease in oAECs of both categories when differentiated towards all three cell lineages (Fig. 4a). The *NANOG* mRNA levels in oAECs obtained from early gestational amnia were exclusively reduced after adipogenic differentiation (*p* < 0.01) (Fig. 4b). By contrast, this gene was always down-regulated when oAECs derived from late amnia were exposed to all three differentiation media (Fig. 4b). Both the *OCT4A* and *SOX2* mRNA expression were reduced in early oAECs only after adipogenic differentiation (Fig. 4c and d); differently, after both osteogenic and neurogenic differentiations of late oAECs the mRNA levels of *OCT4A* were substantially unaltered, while the mRNA content of *SOX2* significantly increased (Fig. 4c and d).

### Effect of oAECs gestational stage on in vitro adipogenic, osteogenic and neuronal differentiation

#### In vitro adipogenic differentiation

**Fig. 5:**
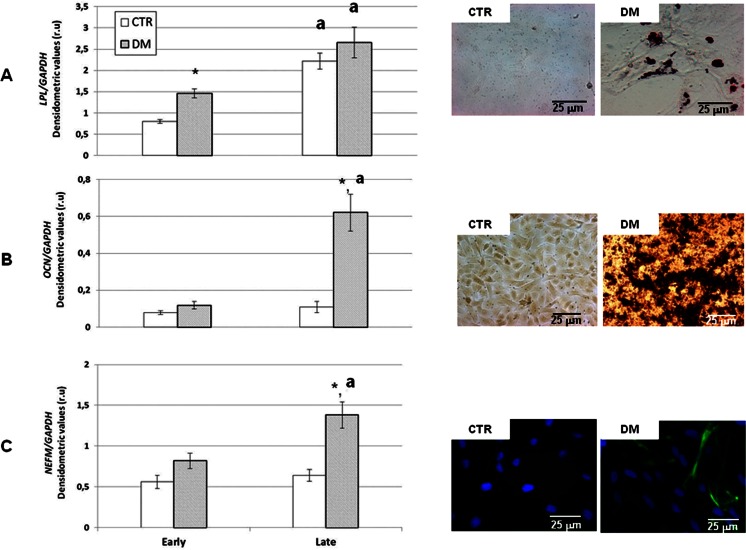
The in vitro differentiation of oAECs obtained from early and late gestational oAECs into adipogenic, osteogenic, and neurogenic cell lineages. *On the left*, the mRNA expression profiles of the tissue specific genes are shown; *on the right*, image examples showing oAECs cultured in growth medium (*CTR*) or in tissue inductive media (*DM*). **a** Adipogenic differentiation: increased expression of the *LPL* mRNA content especially in early oAECs. Images show intracellular lipid droplets in differentiated oAECs after oil red O staining. Scale bar for CTR image and DM image = 25 μm. **b** Osteogenic differentiation: only late oAECs significantly increased the mRNA level of *OCT4A* after differentiation. Extracellular mineralization is documented on the right by alizarin red S staining. Scale bar for CTR image and DM image = 25 μm. **c** Neurogenic differentiation: the *NEFM* mRNA levels significantly increased in oAECs isolated from late amnia. In the right images, NF200-positive cells (*green fluorescence*) demonstrate the neurogenic differentiation of oAECs (nuclei were counterstained with DAPI, *blue*). Scale bar for CTR image and DM image = 25 μm. ^*^ significantly different values between freshly and differentiated oAECs calculated within each category of cell for *p* < 0.05; ^a^ significantly different values between early and late gestational age oAECs calculated within a similar cell category for *p* < 0.05

Both oAECs categories developed vacuoles containing lipid droplets after 30 days of culture performed under adipogenic-inductive conditions (Fig. 5a). However, the vacuoles started to appear earlier in oAECs isolated from late amnia (~4 vs ~10 days in oAECs isolated from late and early amnia, respectively). Freshly isolated oAECs collected from late amnia (Fig. 5a) displayed significantly higher basal levels of *LPL* mRNA, an adipose-related gene. In all analyzed cells, *LPL* mRNA content increased after the adipogenic differentiation, although it became significantly higher only in those oAECs collected from early gestational age amnia (Fig. 5a).

#### In vitro osteogenic differentiation

Both categories of cells were able to mineralize the extracellular matrix, as demonstrated by Alizarin Red staining (Fig. 5b). However, only the oAECs isolated from term amniotic membranes significantly increased the *OCN* mRNA level when incubated under osteo-inductive cultural conditions (Fig. 5b).

#### In vitro neuronal differentiation

The percentage of NF200-positive cells significantly increased (from ≤5 to ~45 %; Fig. 5c) when the oAECs were incubated under neuronal-inductive cultural conditions, independently from cell gestational age. Most of NF200-positive cells, in parallel, acquired an elongated shape.

All the categories of freshly isolated oAECs displayed basal and similar low transcriptional levels of *NEFM,* a neuronal-related gene (Fig. 5c). Its mRNA content increased after 30 days of incubation performed under neuronal-inductive cultural conditions, although this increase resulted to be significant only in oAECs isolated from late amnia (Fig. 5c).

### Effect of oAECs in vitro differentiation on phenotype, genotype and pluripotency markers expression

**Fig. 6:**
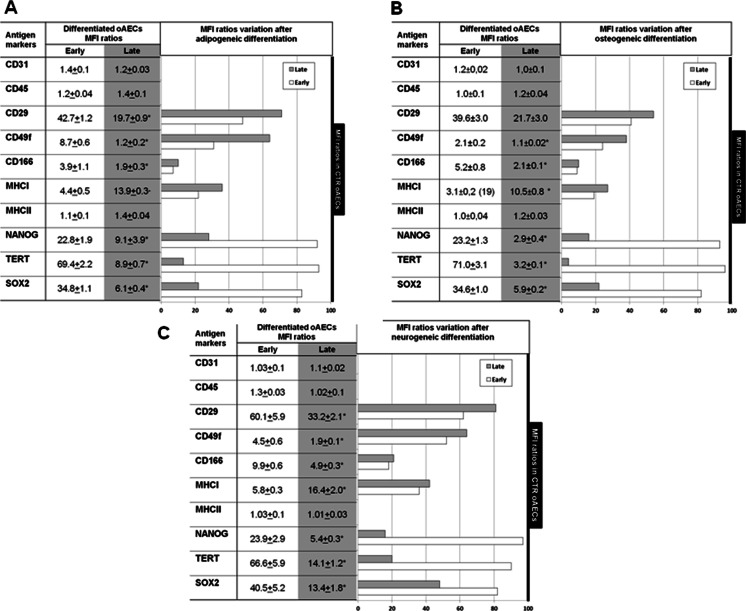
**a–b–c** MFI ratios variation of the analyzed oAEC antigens after in vitro differentiation. On the left of each image, MFI Ratio analyzed by flow-cytometry was calculated in early and late amnia-derived oAECs after adipogenic (**a**), osteogenic (**b**), and neurogenic (**c**) differentiation. * indicates significantly different values between early and late gestational amnia-derived oAECs (*p* < 0.05). On the right, MFI ratio variations are illustrated before and after in vitro differentiation. To compare antigens expression variation, data obtained from freshly isolated oAECs were normalized to 100 % (visualized by the *black bar*). These values were then compared to MFI ratios obtained after the in vitro adipogenic (**a**), osteogenic (**b**) and neurogenic (**c**) differentiation in early and late oAECs. All in vitro differentiations slightly affected stemness markers MFI ratios (NANOG, TERT, SOX_2_) of oAECs isolated at early stages of gestation, while these values were drastically reduced in oAECs collected from late amnia. Adhesion molecules and MHCI MFI ratios were reduced after in vitro differentiation, particularly, in early oAECs

The MFI ratios of stemness markers were unaffected after in vitro differentiation of oAECs isolated at early stage of gestation. Conversely, they dramatically lowered in oAECs collected from amnia of late pregnancies, independently from the cultural conditions adopted (*p* < 0.05) for all pluripotency markers (Fig. 6a–c).

All the differentiated oAECs reduced the MFI ratios of the adhesion molecules and, in particular, of CD166 (*p* < 0.05 for both cell categories: Fig. 6a–c). However, the percentage of this reduction was always higher in differentiated oAECs obtained from amnia at early stage of gestation (*p* < 0.05) (Fig. 6a–c).

A dramatic reduction in MHC I expression was also observed in both cell categories, regardless of the in vitro differentiation cultural conditions used (*p* < 0.05: Fig. 6a–c).

The immunocytochemistry analysis allowed to verify that, after all the differentiation cultural conditions adopted, the percentage of cells that retained a nuclear localization of the pluripotency associated markers was very low in oAECs isolated from late amniotic membranes (≤10 %).

By contrast, oAECs collected from early gestational age amnia after adipogenic differentiation showed a drastic decrease of the percentage of pluripotency molecules within the nucleus (~30 % for NANOG and SOX2 and ~45 % for TERT; data not shown). Differently, the nuclear distribution of these three markers was slightly affected after ostegenic and neurogenic differentiation (data not shown).

Adipogenic differentiation induced in oAECs isolated from early gestation amnia a significant decrease of *NANOG, TERT, SOX2* and *OCT4A* mRNA contents, while in oAECs isolated from late amnia only *TERT* and *NANOG* mRNA levels were reduced (Fig. 4a–d).

Osteogenic and neurogenic differentiations induced in both cells categories a significant decrease in *TERT* mRNA content, while negatively affected *NANOG* expression only in oAECs isolated from late amnia (Fig. 4a–b). In addition, osteogenic and neurogenic differentiations performed in oAECs isolated from late amnia significantly increased the mRNA levels of *SOX2,* while they did not affect the *SOX2* mRNA content in early gestational age oAECs (Fig. 4d).

### Influence of in vitro differentiation on oAECs global DNA methylation

**Fig. 7:**
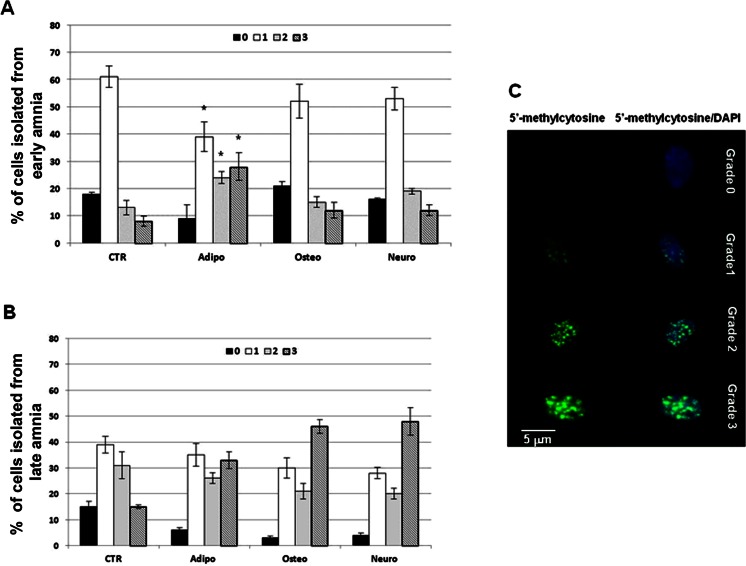
Global DNA methylation analysis performed on both categories of freshly isolated or differentiated oAECs towards the adipogenic, osteogenic or neurogenic lineages. **a**, **b** The graphs show the percentage of the different grades of global DNA methylation in fresh (*CTR*) or differentiated oAECs obtained from amnia at early and late stage of gestation, respectively. Control oAECs isolated from early-stage amnia showed lower global DNA methylation levels than oAECs obtained from late amnia. Early oAECs differentiated toward the adipogenic tissue lineage decreased the percentage of grade 1 cells while increased the incidence of cells with grade 2 and 3. Late oAECs became ipermethylated after all the in vitro differentiation conditions by increasing the percentage of cells with grade 3. * indicates significantly different percentage of grade values calculated between each cell categories (*p* < 0.05). **c** Representative images that show, *on the left*, oAECs 5-methylcytosine positivity (*spotted signals of green fluorescence* distributed within the nucleus). *On the right*, the relative merged images obtained with the simultaneous nuclear staining performed with DAPI (*blue fluorescence*) are shown. The different grades of global DNA methylation were determined by the TFI calculated on single nuclei. On the basis of the TFI emitted by each nucleus, 4° of fluorescence intensities were recognized (i.e., from 0 = no TFI to 3 = maximum TFI). Scale bar = 5 μm

The degree of global DNA methylation increased with gestation. More in detail, ~80 % of freshly oAECs (CTR) isolated from amniotic membranes at early stage of gestation displayed a grade 0–1, while ~70 % of oAECs (CTR) isolated from late amnia had a grade 1–2 of global DNA methylation (Fig. 7a–c).

Ovine AECs isolated from amnia at early stage of gestation changed their status of global DNA methylation exclusively when in vitro differentiated towards the adipogenic cell lineage (Fig. 7a). In this case, the percentage of cells with grade 1 (~40 %: *p* < 0.05) significantly decreased, while increased the percentage of cells displaying the grade 2 (~25 %: *p* < 0.05) and 3 (~30 %: *p* < 0.05). By contrast, the status of methylation remained substantially unchanged when oAECs isolated from amnia at early stage of gestation were cultured under osteogenic or neurogenic-inductive conditions (Fig. 7a).

The percentage of ipermethylated cells increased when oAECs isolated from late amnia were cultured under in vitro differentiation conditions, independently from the cell lineage considered.

More in detail, all the conditions of in vitro differentiation (adipogenic, osteogenic and neurogenic) determined a decrease in the percentage of grade 0 cells (*p* < 0.05) and, a parallel, increase of grade 3 ones (*p* < 0.05: Fig. 7b).

## Discussion

Amniotic membrane and amniotic derived cells have attracted increasing attention in recent years as a possible source of stem cells that may be useful for clinical application in regenerative medicine [2, 19, 41–43]. However, few studies to date have clarified whether the gestational stage of isolation may affect the differentiation potential of AECs [31, 44]. The results obtained in the present study show that sheep AECs greatly changed their phenotype, stemness properties, as well as, their in vitro plasticity during gestation, while they maintained unaltered the immunomodulatory activities.

In fact, one interesting feature of AECs is their immunomodulatory capacities, by suppressing mitogen stimulated T cell proliferation and by modulating macrophage function [45, 46]. Furthermore, these cells transplanted into disease models of lung and liver fibrosis contrast the inflammatory and fibrotic response [47]. In this study, it has been shown that oAECs, independently from the gestational stage of origin, induce a clear down-regulation of lymphocytes proliferation either at passage 1 and 3. The only difference observed was a lower efficiency of expanded (P3) late oAECs to inhibit PBMCS proliferation at high dilution, under cell-to-cell contact test. Both categories of oAECs at passage 3 displayed a lower effect in comparison with oAECs at passage 1, suggesting that in vitro culture might reduce either the inhibitory ability or the number of the cells with immunomodulatory action, as suggested by previous studies using human AECs [45]. Our results show that immunomodulatory capacity is kept both when oAECs are cultured in a cell-to-cell contact with PBMNCs or when they are separated by a transwell system. This result reinforces the hypothesis, extensively studied in human cells [34, 48], that bioactive molecules released from these cells are responsible of the immunosuppressive ability and are playing a fundamental paracrine role in regenerative medicine.

In contrast to the unaffected immunomodulatory properties, other crucial morphological and functional parameters resulted highly affected by cell gestational stage.

Similarly to cells isolated from umbilical cord blood (UCB) collected from premature neonate [49, 50] and to hAECs [44], oAECs obtained at early stage of gestation displayed a higher proliferative activity. As supposed for UCB derived cells, this aspect could be related to the larger amount of stem/progenitor cells, as well as, to a more rapid cell cycle required in preterm fetuses in order to drive an intense organogenesis [50]. The hypothesis of a larger aliquots of stem/progenitor cells in amniotic derived cells obtained from early amnia could be suggested by the higher expression of pluripotency markers, but not by the in vitro differentiation experiments. In fact, oAECs isolated in earlier stage of gestation displayed the ability to differentiate toward the adipogenic lineage, while they were unable to undergo to a full osteogenic differentiation.

Adipogenic plasticity was clearly confirmed in oAECs isolated at early gestation by evaluating the expression of *LPL,* a specific gene that becomes transcriptionally active when pre-adipocytes differentiate into adipocytes [51]. Its mRNA content became significantly higher in this type of preterm oAECs during the in vitro adipogenic differentiation, as previously observed in human [52] and chicken [53] AECs. Interestingly, *LPL* mRNA content revealed that gestation determined an adipogenic drift in both control and differentiated amniotic derived cells, thus revealing their spontaneous mesodermal transformation. On the contrary, oAECs derived from term amnia seem to have a greater plasticity toward both osteogenic and neurogenic cell lineage differentiation, when compared to non-differentiated cells.

In the present research, the in vitro osteogenic potential of oAECs was further confirmed [3, 21], although this plasticity was shown to be strictly related to the time of gestation. In fact, while osteogenic cell evolution appeared to be easily obtained by incubating oAECs isolated from mid [3, 21] to term gestation amnia, oAECs collected from early gestation amnia have proved to be highly refractory to the osteogenic-inductive cultural condition.

In parallel, term placenta-derived oAECs displayed a neurogenic plasticity, similarly to the one already demonstrated in human AECs [15]. The neurogenic attitude of oAECs was tested by combining the addition of FGF and retinoic acid in cultural medium, that previously have been shown to improve AEC neuronal markers expression [15], compared to the use of single inductive factors [54]. Also for in vitro neurogenic differentiation, neither the morphological neuronal phenotype or the intracellular accumulation of NF200 protein were able to document a different attitude between oAECs of different gestational age. On the contrary, a different predisposition was confirmed by the expression of a specific neurofilament gene (*NEFM)* that was more expressed in oAECs isolated from term fetuses.

Altogether, these evidences demonstrated that oAECs underwent to a functional transformation during pregnancy. This conclusion has been previously suggested by Lim et al. [44] that showed as cells derived from preterm amnion are less effective in terms of protection against lung inflammation and fibrosis, than those collected from amnion at the end of gestation. All these results suggest a distinctive clinical impact after their autologous administration.

In the present study, an additional conclusion can be gained. In fact, oAECs derived from early and late amnia seem to display a distinctive in vitro differentiation attitude, due to their different gestational age. Indeed, Lim and colleagues previously showed that while preterm hAECs were able to express both adipose and neural markers, they were less able to express lung lineage markers than term cells [44]. Combining the data obtained, it could be hypothesized that AEC differentiation potential is different throughout gestation, and that these differences may be attributable to both gestation and considered cell lineages. This conclusion is either supported by the experiments of in vitro differentiation and, in particular, by the expression analysis of *NANOG*, that exactly reproduced what has been previously documented in embryonic stem cells (ES) by Hyslop et al. [55]. In fact, the content of *NANOG* mRNA resulted to be reduced only in the oAECs that underwent in vitro differentiation; indeed, *NANOG* levels dropped in oAECs isolated from early stage amnia after their adipogenic differentiation, while the levels remained unaltered following the incubation performed under osteo- or neuro-inductive conditions. Differently, the decrease in *NANOG* mRNA content in oAECs isolated from term amnia was higher after osteogenic and neurogenic differentiation than that observed after the adipogenic one.

Another important difference observed between the two categories of oAECs was the expression of the pluripotency markers that may suggest the existence and persistence after the in vitro differentiation of different rates of stem/progenitor cells. In fact, the basal expression of *TERT*, *SOX2* and *NANOG* resulted to be significantly higher in oAECs isolated from amnia at early stage of gestation and their expression remained at high levels also after the adipogenic differentiation. The finding that the expression of both *NANOG* and *SOX2* mRNAs were significantly higher in the early gestational oAECs than in the late ones is concordant to the results reported in fetal and term human AECs [31].

The absence of TERT mRNA observed by Miki et al. [15] in human term AECs could be explain by a progressive switch off of this gene during the gestation more than to a different specie-specific behavior. Also, Nakajima et al. [30] had previously reported that telomerase activity was higher in rat AECs isolated from mid stage (day 13.5 to 15.5) amnia than in the late stage (day 17.5 to 21.5) ones. Concordant to the result reported by Izumi et al. [31] in human AECs, OCT4 mRNA expression was not affected by gestation. The persistence of this stem cells marker during the course of pregnancy might be the consequence of a symmetrical cell cleavage that could guaranty that term amniotic derived cells retain the epiblast-derived genotype [56, 57].

A functional difference between the two categories of oAECs was also suggested by the cellular localization and the levels of expression of pluripotency markers after the in vitro process of differentiation. In fact, while oAECs significantly reduced the expression of the adhesion molecules, the expression of pluripotency markers remained quite stable in oAECs isolated from amnia at early stage of gestation following differentiation. This result may be a signal of stemness persistence in the differentiated early gestational age oAECs that is also supported by the higher percentage of cells displaying the pluripotency markers within the nucleus. The presence in the nucleus of the analyzed embryonic specific factors and their persistence in this cellular compartment is critical for the maintenance of stemness features, while the absence of expression in the nucleus of even a single factor may impair cell pluripotency. Indeed, in this research the nuclear distribution of pluripotency markers suggests that oAECs isolated from term amnia show signals of stemness weakening already immediately after their isolation, and that this decline further worsen after in vitro differentiation.

A different behavior between the two categories of oAECs was also observed by analyzing the expression profile of pluripotency markers. The adipogenic differentiation induced an overall down-regulation of pluripotency related genes in oAECs derived from amnia at early stage of gestation, differently from the ones isolated from term amniotic membranes.

Interestingly, *TERT* mRNA content underwent to a generalized decrease when oAECs were exposed to differentiation medium, independently from the ability of the cultural conditions to enable the relative cell-lineage differentiation. In fact, a constant switch-off in the transcriptional activity of *TERT* was observed in both categories of oAECs, thus demonstrating that this genes is not strictly related to the process of in vitro differentiation, as previously demonstrated in embryonic stem cells [58, 59]. The drastic down-regulation of *TERT* expression in differentiated oAECs exposed the cells to a condition of proliferative senescence that, however, could be less effective in early oAECs, in which ~45 % of cells maintained TERT confined within the nucleus, indicating a tardive protein degradation.

Finally, in this study, global DNA methylation was defined in fresh and differentiated oAECs. This analysis showed a progressive increase in the global DNA methylation status revealing an epigenetic status that was not stable throughout gestation. In fact, the degree of global DNA methylation was positively related to the status of differentiation in mouse and human embryonic stem cells [60, 61], in which the DNMT deficiency, that reduced the levels of methylation, was also able to block their differentiation potential [62].

The lower methylation level observed in oAECs isolated from early stage amnia could represent, therefore, another evidence of their higher stemness feature that diminishes once these cells undergo to the adipogenic differentiation. The in vitro differentiation that determined a molecular cell-lineage transformation was, in fact, able in oAECs to increase the percentage of ipermethylated cells (with grade 3). This feature became particularly relevant in oAECs derived from term amnia exposed for 30 days to osteo and neuro-inductive conditions.

Although the functional role of global DNA methylation remains to be clarified, the present results confirm that oAECs derived from early stage of gestation displayed before and after the in vitro differentiation a more homogenous and a lower global DNA methylation, thus suggesting their higher stemness property, as previously suggested by phenotype and genotype analyses.

In conclusion, this study confirms that oAECs modified their phenotype, stemness and plasticity during gestation, thus suggesting an evolution of amnion. This results seem to open an applicative consequence: cells gestational age could, in fact, supports different mesodermal and endodermal lineages regenerative properties, as well as, it may offer the transplantation of different rates of stem/progenitor cells. In this context, it is interesting to recall that amnion-derived cells have been proposed as a superior cell type for the production of induced pluripotent stem cells (iPSC) [63–66]. However, only in vivo experiments will be able to demonstrate the full potential regenerative features of this type of stem cells, as suggested by preclinical studies carried out in different transplantation settings [9, 10, 21, 24, 25]. Finally, the higher expression of pluripotency markers combined with the lower global DNA methylation reported for early gestational age oAECs may offer favorable biological conditions for all those approaches that involve nuclear reprogramming, such as the somatic cell nuclear transfer (SCNT) and the production of induced pluripotent stem cells (iPSC).
